# Deciphering the functions of Stromal Interaction Molecule-1 in amelogenesis using AmelX-iCre mice

**DOI:** 10.3389/fphys.2023.1100714

**Published:** 2023-03-01

**Authors:** Raed Said, Helyasadat Mortazavi, David Cooper, Katie Ovens, Ian McQuillan, Silvana Papagerakis, Petros Papagerakis

**Affiliations:** ^1^ College of Dentistry, University of Saskatchewan, Saskatoon, SK, Canada; ^2^ Department of Anatomy, Physiology and Pharmacology, College of Medicine, University of Saskatchewan, Saskatoon, SK, Canada; ^3^ Department of Computer Science, University of Calgary, Calgary, AB, Canada; ^4^ Department of Computer Sciences, College of Arts and Sciences, University of Saskatchewan, Saskatoon, SK, Canada; ^5^ Department of Surgery, College of Medicine, University of Saskatchewan, Saskatoon, SK, Canada; ^6^ Department of Otolaryngology-Head and Neck Surgery, School of Medicine, University of Michigan, Ann Arbor, MI, United States

**Keywords:** enamel, calcium, ameloblast, amelogenesis, stromal interacting molecule 1 (STIM1), store operated Ca 2+ entry, mineralised tissue

## Abstract

**Introduction:** The intracellular Ca2+ sensor stromal interaction molecule 1 (STIM1) is thought to play a critical role in enamel development, as its mutations cause Amelogenesis Imperfecta (AI). We recently established an ameloblast-specific (AmelX-iCre) Stim1 conditional deletion mouse model to investigate the role of STIM1 in controlling ameloblast function and differentiation *in vivo* (Stim1 cKO). Our pilot data (Said et al., J. Dent. Res., 2019, 98, 1002–1010) support our hypothesis for a broad role of Stim1 in amelogenesis. This paper aims to provide an in-depth characterization of the enamel phenotype observed in our Stim1 cKO model.

**Methods:** We crossed AmelX-iCre mice with Stim1-floxed animals to develop ameloblast-specific Stim1 cKO mice. Scanning electron microscopy, energy dispersive spectroscopy, and micro- CT were used to study the enamel phenotype. RNAseq and RT-qPCR were utilized to evaluate changes in the gene expression of several key ameloblast genes. Immunohistochemistry was used to detect the amelogenin, matrix metalloprotease 20 and kallikrein 4 proteins in ameloblasts.

**Results: **Stim1 cKO animals exhibited a hypomineralized AI phenotype, with reduced enamel volume, diminished mineral density, and lower calcium content. The mutant enamel phenotype was more severe in older Stim1 cKO mice compared to younger ones and changes in enamel volume and mineral content were more pronounced in incisors compared to molars. Exploratory RNAseq analysis of incisors’ ameloblasts suggested that ablation of Stim1 altered the expression levels of several genes encoding enamel matrix proteins which were confirmed by subsequent RT-qPCR. On the other hand, RT-qPCR analysis of molars’ ameloblasts showed non-significant differences in the expression levels of enamel matrix genes between control and *Stim1*-deficient cells. Moreover, gene expression analysis of incisors’ and molars’ ameloblasts showed that *Stim1* ablation caused changes in the expression levels of several genes associated with calcium transport and mitochondrial kinetics.

**Conclusions:** Collectively, these findings suggest that the loss of *Stim1* in ameloblasts may impact enamel mineralization and ameloblast gene expression.

## Introduction

Dental enamel is the most calcified tissue in the body serving as the protective outer layer for teeth withstanding masticatory forces (Nanci, 2013a). Enamel formation, amelogenesis, is a complex process that results in the development and mineralization of dental enamel (Simmer et al., 2010; Zheng et al., 2014). Amelogenesis is carried out by ectodermally derived epithelial cells of the enamel organ, termed ameloblasts. Amelogenesis presents in two main developmental stages known as the secretory and maturation stages (Simmer et al., 2021). During the secretory stage, ameloblasts provide a proteinaceous template for enamel crystal growth, which essentially mineralizes during the maturation period (Paine et al., 2020). The functional characteristics of ameloblasts are tightly linked to their differentiation stage which confers them to a specific morphology. Indeed, during the maturation stage, ameloblasts cyclically modulate their morphology between a ruffle-ended (RA) appearance and a smooth-ended (SA) appearance which may reflect the alternation of the functional phase between resorptive and secretory (Nanci, 2013b; Paine et al., 2020).

Ca2+ is one of the most important ions in enamel, as it participates in the formation and growth of enamel crystals (Iijima et al., 2002). Indeed, enamel hydroxyapatite-like crystals form *de novo* by precipitating ions in the enamel space after passing through the semipermeable barrier created by ameloblasts (Lacruz, 2017). During crystal formation, the ameloblasts transfer large quantities of calcium from the serum into the enamel space. Approximately 60% of the minerals in the fully developed enamel are supplied during the maturation stage (Kirkham et al., 1988). It has been initially proposed that calcium transport occurs in two primary ways: paracellular and transcellular (Berdal et al., 1995; Bailleul-Forestier et al., 1996; Hubbard, 2000; Papagerakis et al., 2002). Several subsequent reports, however, showed that Ca2+ is primarily transported through high-capacity stores associated with the endoplasmic reticulum (ER), widely distributed in ameloblasts, suggesting that the store-mediated transcellular calcium transport is the dominant mode of ion transport across the ameloblast (Lacruz et al., 2012; Nurbaeva et al., 2015a). Impaired ion transport across the ameloblast may result in the hypomineralization of enamel (Lacruz and Feske, 2015; Silva-Rojas et al., 2020).

Store-operated Ca2+ entry (SOCE) has been established as a major Ca2+ influx mechanism in excitable cells and non-excitable cells (Jardín et al., 2009). Depletion of Ca2+ in the ER triggers SOCE, which entails the formation of multimers of stromal interaction molecule 1 (STIM1), an ER transmembrane protein, that is translocated to the ER-plasma membrane junction (Feske, 2009; Smyth et al., 2010). Ca2+ release-activated Ca2+ channels such as ORAI1 are opened by STIM1 once it binds to it, leading to sustained Ca2+ influx (Li et al., 2007). The STIM2 family member is also located in the ER and acts as a positive regulator of SOCE, and its functions partially overlap with STIM1 as they share >60% sequence identity (Berna-Erro et al., 2017). By allowing Ca2+ to influx into the cytoplasm by ORAI1, the cytosolic Ca2+ concentration increases and the ER Ca2+ stores are replenished *via* sarcoplasmic/ER Ca2+-ATPases (SERCAs) (Eckstein and Lacruz, 2018). Several studies showed that both *Stim1* and *Stim2* are robustly expressed in ameloblasts during their secretory and maturation stages in wild-type rodents, suggesting a crucial role for SOCE in the influx of calcium into ameloblasts (Lacruz et al., 2011; Lacruz et al., 2012; Nurbaeva et al., 2015a; Nurbaeva et al., 2015b).

Ectodermal dysplasia is characterized by defects in several ectodermal tissues, including hair, nails, sweat glands and dental enamel causing amelogenesis imperfecta (AI) (Itin and Fistarol, 2004; Furukawa et al., 2017). Based on several previous studies, patients with mutations in *STIM1* and *ORAI1* exhibit ectodermal dysplasia with amelogenesis imperfecta, which further confirms the significance of SOCE in enamel mineralization (Feske et al., 2010; Lacruz and Feske, 2015; Silva-Rojas et al., 2020). Indeed, several loss-of-function (LoF) mutations in *STIM1* and *ORAI1* have been hitherto reported in the literature that affect enamel quite severely in both primary and permanent dentition where the severely hypomineralized enamel wears extremely rapidly (Picard et al., 2009; Wang et al., 2014; Lacruz and Feske, 2015; Lian et al., 2018). Moreover, it has been demonstrated that patients with mutations in *STIM1* or *ORAI1* exhibit a severe immune impairment (Picard et al., 2009; Lian et al., 2018).

*In vitro* experiments with ameloblast-like cells suggest that the expression of several enamel proteins encoding genes may be regulated by SOCE (Nurbaeva et al., 2015b; Zheng et al., 2015; Eckstein et al., 2019). The mechanisms by which SOCE regulates amelogenesis *in vivo* were greatly understudied as deletion of *Orai1, Stim1*, or both *Stim1/Stim2* genes results in perinatal lethality in mice, making the effective animal models with global impaired SOCE of limited use (Said et al., 2019). To address this issue, we and others have recently established ectodermal specific (i.e., Keratin14 (K14)-Cre mediated) and ameloblast-specific (AmelX-iCre) mice models where *Stim1, Stim2,* and *Orai1* genes and some of their combinations were ablated in ameloblasts to investigate the roles of SOCE in controlling ameloblast function and differentiation *in vivo* (Eckstein et al., 2017; Furukawa et al., 2017; Eckstein et al., 2019; Said et al., 2019). All of these abovementioned mice show reduced or absent SOCE in ameloblasts and have an AI phenotype with hypomineralized enamel. The main advantage of the AmelX-Cre line used here over the K14 Cre line is its ability to target gene deletion specifically in ameloblasts from the secretory stage onwards while K14-Cre line deletes genes in all layers of the enamel organ: ameloblasts, stratum intermedium and papillary layer (Klein et al., 2017).

Pilot data from our ameloblast-specific (AmelX-iCre) *Stim1* conditional deletion mouse model (Stim1 cKO) suggest a wide role of STIM1 in amelogenesis (Said et al., 2019). This paper aimed to provide a further in-depth characterization of *Stim1* mutant incisors and molars in this Stim1 cKO model. We hypothesized that amelogenesis imperfecta due to abnormal SOCE may be caused by changes in the enamel mineralization process due to altered ion transport across the ameloblast cell layer and changes in the expression of enamel matrix proteins and their proteases. To test these hypotheses, we ablated the *Stim1* gene from enamel-forming ameloblasts by crossing *Stim1* floxed mice with Amelogenin–iCre (AmelX-iCre) transgenic mice and analyzed their enamel phenotype and ameloblasts genetic profile. Our analysis showed that the expression of *Stim1* in the enamel cells of the knock-out mice was significantly reduced. These mice were found to have severely hypomineralized and thin enamel that wore rapidly, with lower calcium levels, all of which were similar to the human dental AI phenotypes. The mutant enamel phenotype was more severe in older Stim1 cKO mice compared to younger ones and changes in enamel volume were more pronounced in incisors compared to molars. Our gene expression analysis and suggests that ablation of *Stim1* affected the expression levels of the genes encoding enamel matrix proteins in incisors’ ameloblasts more considerably than in molars’ ameloblasts. Furthermore, data from our gene expression analysis of incisors’ and molars’ ameloblasts suggest that *Stim1* invalidation altered the expression of several genes encoding other calcium pumps and exchangers involved in calcium transport during amelogenesis in addition to genes associated with the cell mitochondrial kinetics and bioenergetics. Collectively, our results suggest that SOCE mediated by STIM1 is important for both the mineralization of dental enamel as well as the regulation of gene expression in ameloblasts, and thus the overall functioning of the cell.

## Materials and methods

### Generation of Stim1 cKO mice

All mice were housed in a specific pathogen-free facility and fed *ad libitum* with a normal diet and sterilized water under a periodic light/dark cycle. AmelX-iCre transgenic mice (Said et al., 2020) and Stim1^fl/fl^ mice (Oh-hora et al., 2008) were mated and the progeny bred to generate the Stim1 cKO mice (AmelX-iCre^+^; Stim1^fl/fl^) mice. AmelX-iCre^-^; Stim1^fl/fl^ mice were used as a control genotype. Genotypes were determined by tail biopsy under 3% isoflurane and by conventional polymerase chain reaction (PCR) as previously reported (Said et al., 2019). Only male mice with the desired genotypes were euthanized by an overdose of carbon dioxide followed by cervical dislocation for subsequent analyses. All animal procedures and breeding programs were approved by the University of Saskatchewan Animal Care and Use Committee and the Animal Research Ethics Board (protocol # 20170014).

### Dissecting microscopy

The mandibles were removed and dissected free of soft tissues then fixed by immersion in 10% neutral buffered formalin (NBF). The teeth were cleaned with non-woven gauze, displayed on the Nikon SMZ1000 (Nikon Corporation, Japan) dissection microscope and photographed using a Nikon digital camera DXM1200 (Nikon Corporation, Japan) for gross morphometric analysis.

### Scanning electron microscopy and energy dispersive spectroscopy

Scanning electron microscope (SEM) evaluation was performed at the Western College of Veterinary Medicine Imaging Centre at the University of Saskatchewan (Saskatoon, Canada). Ethanol-dehydrated and air-dried samples from the control and Stim1 cKO mice were mounted on metallic stubs using conductive carbon cement and degassed in a vacuum desiccator overnight. The samples were imaged using a Hitachi SU8010 Scanning Electron Microscope (Hitachi High-Tec, Tokyo, Japan) operating at an accelerating voltage of 3.0 kV. The Hitachi SU8010 is a semi-in-lens type cold field emission that offers backscattered electron (BSE) detection and energy dispersive spectroscopy (EDS) for compositional information using the AztecLiveStandard with Ultim Max 170 Detector. For BSE incisor imaging, the bony caps and soft tissue covering the mandibular incisors were carefully removed and the labial surface of the mandibular incisors was then examined at × 150 magnification in a Hitachi SU8010 scanning electron microscope using the backscatter mode. Gray levels of the BSE images were measured using the ImageJ software. For EDS, 4 beams were generated on the cusp slopes of maxillary first molars that received no processing of the enamel surface in order not to alter its composition. Comparisons were made using a total of *n* = 3 mice per group. The mandibular incisors were sectioned using a diamond saw at their site of eruption on the level of the alveolar bone crest; this level was used to assess enamel thickness relative to dentin. Thickness was measured using the ImageJ software. Mandibular first molars were sagittally sectioned at the level of mesiobuccal cusp; this level was used to assess the prismatic structure under the SEM. The molar samples were cleaned by brushing in soapy water for at least 1 min, using a medium-to-stiff and dense artist’s brush, then were put in a jar with distilled water in an ultrasonication basin for about 5 min. The samples were then polished at the following grits: 600, 800 and 1200, then they were etched with nitric acid of various concentrations (0.1%, 0.5%, 1%, 2.5%, and 5%), all prepared with distilled water (Risnes et al., 2019) and sputter coated with gold prior to imaging.

### Micro–computed tomography

Hemimandibles (weeks 2, 4, 12, *n* = 3 per each genotype) were dissected, fixed for 24 h in NBF, and stored in 70% ethanol at 4°C. Hemimandibles were then scanned with a micro–computed tomography (μCT) scanner (6.7 μm isotropic voxel size, 50 kVp, 250 μA, Al 0.5 mm filter, 0.2 rotation degree step, 440 ms exposure time; Skyscan1172, Bruker microCT, Massachusetts, United States). After 2-dimensional reconstruction with NRecon the data were imported in Amira software (https://www.fei.com) for 3D evaluation. The image data were segmented employing semiautomatic tools in order to separate the enamel from the dentine and generate 3D surface models of the teeth. The threshold value for imaging was raised until enamel was the only mineral displayed in the control group and total incisor and molar enamel volumes were measured. For mineral density analysis, incisor images were obtained perpendicular to the long axis of the incisor and parallel to the mesial root canal of the mandibular first molar as this portion of the tooth is not affected by wear yet while being fully mature and already mineralized. The mineral density (gHA/cm3) on the incisor enamel was calculated for quantitative analysis after the calibration with phantoms (SP-4002, Bruker microCT) according to the manufacturer’s instructions.

### Explarotary RNAseq and data processing

Whole incisor enamel organ (EO) cell populations of P28 old mice were carefully dissected, and total RNA was isolated using the RNeasy Micro Kit (Qiagen) from 3 samples (*n* = 1 per genotype); 1 Stim1 cKO sample (Stim1 cKO) with complete deletion of *Stim1* (AmelX-iCre^+^; Stim1^fl/fl^), 1 Stim1 heterozygous (Stim1 HT) sample with heterozygous deletion of one *Stim1* allele (AmelX-iCre^+^; Stim1^fl/wt^) and 1 control sample (Stim1 Ctrl) with no *Stim1* deletion as it was Cre negative (AmelX-iCre^-^; Stim1^fl/fl^). RNA quality was analyzed on Agilent Bioanalyzer 2100 RNA Nano chip following Agilent Technologies’ recommendation. Concentration was measured by Qubit RNA HS Assay on a Qubit fluorometer (ThermoFisher). Library preparation was performed at The Centre for Applied Genomics in The Hospital for Sick Children (Toronto, Canada) following the NEB NEBNext Ultra II Directional RNA Library Preparation protocol. Briefly, 400 ng of total RNA was used as the input material and enriched for poly-A mRNA, fragmented into the 200-300-bases range for 4 min at 94°C and converted to double stranded cDNA, end-repaired and adenylated at the 3’ to create an overhang A to allow for ligation of Illumina adapters with an overhang T; library fragments were amplified under the following conditions: initial denaturation at 98°C for 10 s, followed by 15 cycles of 98°C for 10 s, 60°C for 30 s and 72°C for 30 s, and finally an extension step for 5 min at 72°C; at the amplification step, each sample were amplified with a different barcoded adapters to allow for multiplex sequencing. One ul of the final RNA libraries was loaded on a Bioanalyzer 2100 DNA High Sensitivity chip (Agilent Technologies) to check for size; RNA libraries were quantified by qPCR using the Kapa Library Quantification Illumina/ABI Prism Kit protocol (KAPA Biosystems). Libraries were pooled in equimolar quantities and paired-end sequenced on 0.5 lanes of a High Throughput Run Mode flowcell with the V4 sequencing chemistry on an Illumina HiSeq 2500 platform following Illumina’s recommended protocol to generate paired-end reads of 126-bases in length. Quality control of raw sequencing reads was performed using FastQC and low-quality reads were removed. PHRED score and sequence duplication levels were calculated using the Galaxy platform (Jalili et al., 2021). Trimmed paired-end reads were aligned to the mouse genome using STAR ultrafast universal RNA-seq aligner.

To calculate transcript abundances, we converted raw reads to TPM values and differential expression analysis was performed using DESeq2 package. Principal component analysis and distribution analysis were used to identify outlier samples. Differences in gene expression were considered statistically significant if the adjusted *p*-value was less than 0.01 with absolute fold change >2. Enrichment analysis was performed using the gene ontology (GO) found in the Galaxy platform in addition to the PANTHER tool (PANTHER 17.0) for pathway enrichment. Data visualization was done using the DESeq2 and R packages. Heatmaps and clustering of selected genes show the highest and lowest expression for each gene displayed as blue and red (min and max), respectively. Raw and processed data have been deposited in the Gene Expression Omnibus (GEO) repository with the accession number GSE218388 (https://www.ncbi.nlm.nih.gov/geo/query/acc.cgi?acc=GSE218388).

### Tooth RNA extraction and quantitative PCR

Ameloblasts were carefully isolated from the molars and incisors of mice aged between P10 to P12 (n = 3 to 4 per genotype) and used for total RNA extraction (RNeasy, Qiagen). After cDNA synthesis (SuperScript III, Thermo Fisher Scientific), the gene expression levels of amelogenin (*Amelx*), ameloblastin (*Ambn*), enamelin (*Enam*), *Orai1*, *Serca1* (expressed by the *Atp2a1* gene), *Serca2* (expressed by the *Atp2a2* gene), *Serca3* (expressed by the *Atp2a3* gene), Plasma membrane Ca2+ ATPase 1 (*Pmca1*, expressed by the *Atp2b1* gene), Plasma membrane Ca2+ ATPase 4 (*Pmca4*, expressed by the *Atp2b4* gene), Na+/Ca2+-K+ exchanger (*NCKX4*, expressed by the *Slc24a4* gene), mitochondrially encoded ATP synthase membrane subunit 6 (*mt-Atp6*), Mitochondrially Encoded NADH:Ubiquinone Oxidoreductase Core Subunit 1 (*mt-Nd1*) and vimentin (*Vim*) (to exclude pulp cells contamination in molars ameloblasts) were assessed by quantitative polymerase chain reaction (RT-qPCR; Applied Biosystem PowerUp SYBR™ Green Master Mix, Thermo Fisher Scientific, Waltham, Massachusetts, United States) with the 2^−ΔΔCT^ method. Ameloblasts were also carefully isolated from the molars (P14) and incisors (P28) to assess the expression levels of *Stim1* and *Stim2.* Quantification was calculated relative to the housekeeping B*-actin* gene. All primer sequences were obtained from previous publications or Primer Bank (http://pga.mgh.harvard.edu/primerbank/; Supplementary Table S1).

### Immunohistochemistry

Mouse heads were sectioned in a coronal (P5) and sagittal plane (P14). The samples (*n* = 3 to 4 per genotype) were then fixed in neutral buffered formalin (NBF) and demineralized by ethylenediaminetetraacetic acid (EDTA). Paraffin sections (6 μm) were immune stained to assess STIM1, AMLEX, kallikrein 4 (KLK4) and matrix metalloprotease 20 (MMP20) protein cellular and tissue localization using a rabbit Anti-AMELX antibody (1:1000, ab153915), a rabbit Anti-STIM1 (1:200, LSC34692), a rabbit Anti-KLK4 (1:50, PA5-109888) and a rabbit Anti-MMP20 (1:100, ab198815) after antigen retrievals using proteinase K (DAKO, Santa Clara, USA). Primary antibodies were diluted with an antibody diluent containing phosphate-buffered saline (PBS), bovine serum albumin (BSA), and glycerol (DAKO, Santa Clara, United States). Following several washes by PBS + 1% BSA, the sections were incubated for 30 min with a Horseradish Peroxidase (HRP) conjugated goat anti-rabbit secondary antibody (DAKO, Santa Clara, USA). Visualization with 3-3′diaminobenzidine (DAB) was performed using the peroxidase substrate kit DAB (ab64238, Abcam Laboratories, Cambridge, United Kingdom) instructions, and the reaction was stopped after 5 min of exposure. Consecutive serial sections from the same mouse models incubated with rabbit serum instead of primary antibody were used as a control. Sections were then mounted and photographed with an EVOS 5000 microscope.

### Statistics

All data are presented as mean ± SEM. Student’s t-test was used to compare the RT-qPCR relative gene expression, enamel thickness, BSE gray levels and mineral composition. A *p*-value < 0.05 was considered significant.

## Results

### Tissue-specific deletion of Stim1

**FIGURE 1:**
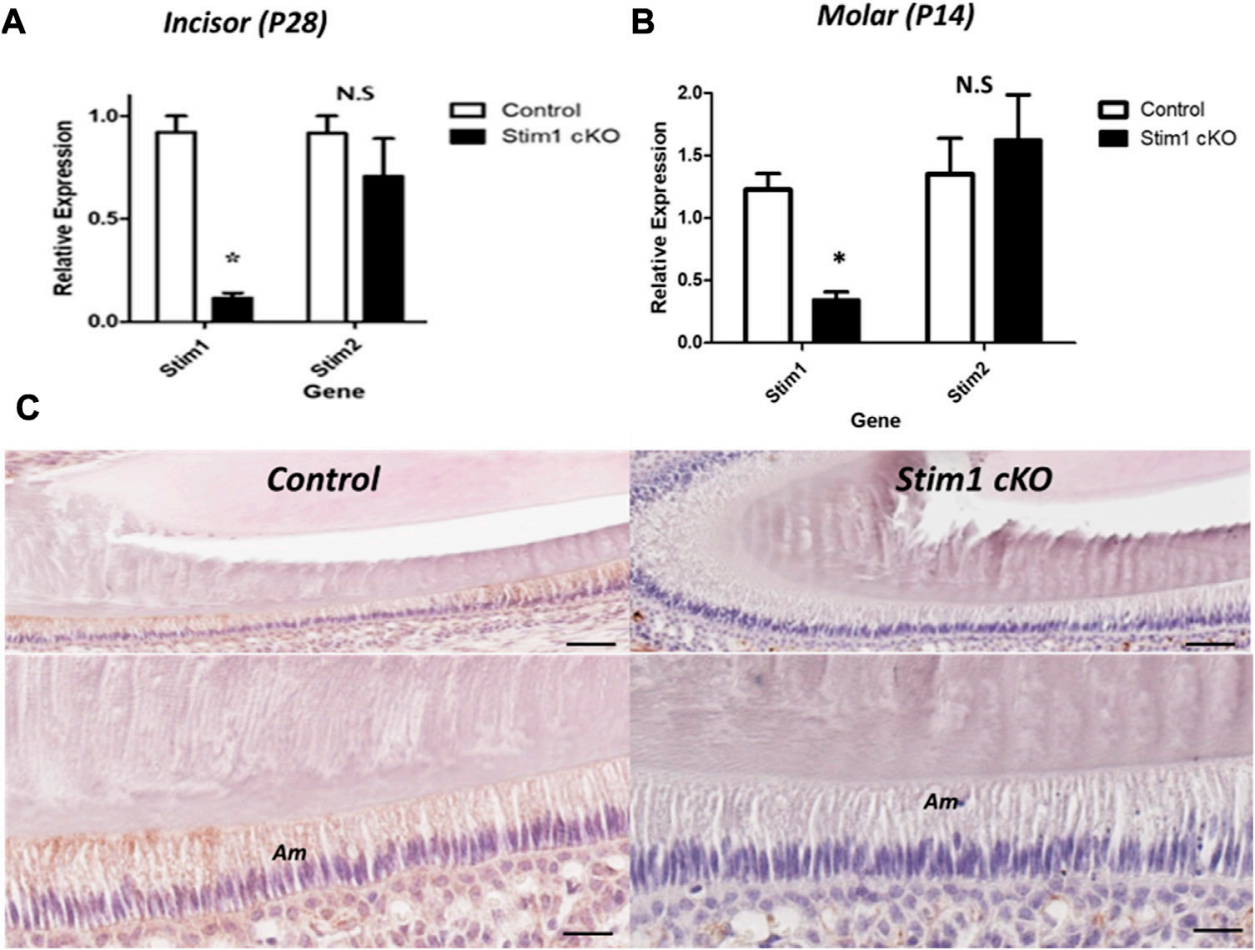
Characterization of Stim1 gene deletion. **(A,B)** RT-qPCR analysis in control and Stim1 cKO ameloblasts collected at postnatal day 28 (P28) from incisors **(A)** and at postnatal day 14 (P14) from molars **(B)** showed that the expression levels of *Stim1* were significantly reduced after *Stim1* deletion while the levels of its analog *Stim2* were not significantly reduced. **(C)** IHC of STIM1 antibody confirmed deletion of STIM1 protein in the ameloblasts of incisor teeth collected at postnatal day 14; STIM1 was robustly expressed in the ameloblasts (Am) in control mice while it was barely detected in *Stim1* conditional knock-out mice. The low mag images of the upper panel are enlarged in the lower panel for each sample. Am, ameloblasts. Scale bars; **(C)** 80 μm in low mag panels, 40 μm in high mag panels. (*p < 0.01), N.S; non-significant.

The expression levels of *Stim1* and *Stim2* mRNA were measured to confirm the conditional deletion of *Stim1* in P14 M and P28 incisors’ ameloblasts. The *Stim1* expression levels were significantly reduced in Stim1 cKO mice (Figures 1A, B). *Stim2* expression showed no significant difference between the genotypes (Figures 1A, B). Immunohistochemical analysis of two weeks old mice ameloblasts was also performed to identify the protein abundance and localization of STIM1. Control mice showed higher STIM1 signals in the maturation stage ameloblasts as compared with those in the secretory stage. Signals for STIM1 were almost undetectable in incisor ameloblasts of Stim1 cKO mice (Figure 1C). In both groups, STIM1 signals were still clearly detected in mesenchymal-derived tissues and non-ameloblast cell populations indicating that *Stim1* was successfully knocked out specifically in enamel-forming cells and the Cre recombinase effect did not leak to adjacent tissues.

### Stim1 cKO mice displayed amelogenesis imperfecta (AI) phenotypes

**FIGURE 2:**
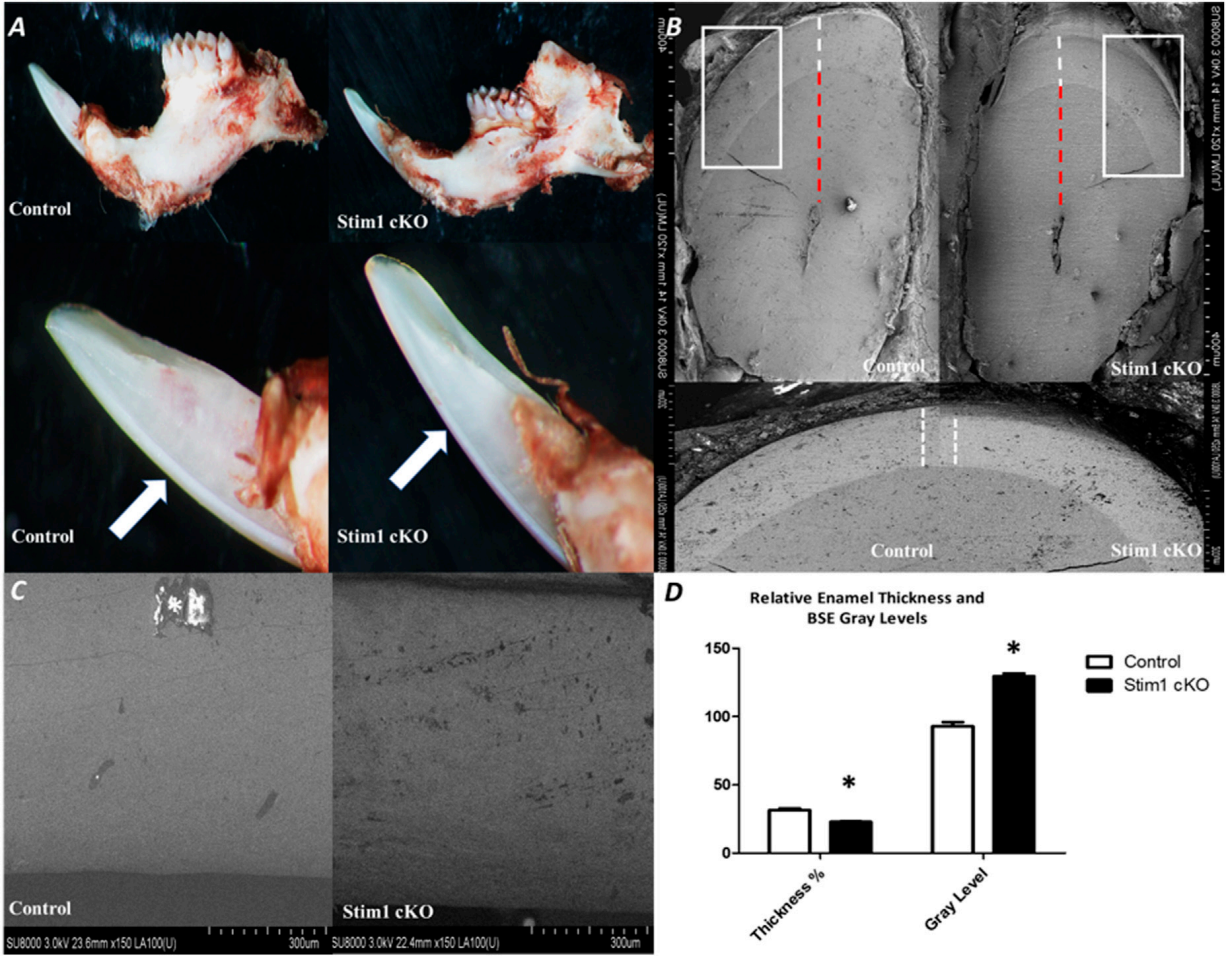
Phenotypic analysis of Stim1 cKO incisors **(A)** Gross visual inspection of the P28 incisors of Stim1 cKO mice showed an abnormal chalky-white appearance that is often associated with hypomineralization or enamel loss. Stim1cKO mice showed a greatly reduced enamel thickness closer to the incisor tips (arrows). **(B)** Stim1 cKO incisor displayed a clearly reduced enamel thickness (white dotted line) as compared with control. For quantitative analysis, we measure the enamel thickness and divided it by the total of enamel and dentin (red dotted line) to attain a more representative measure of enamel thickness relative to the crown. **(C)** Backscatter scanning electron microscopy of mid labial surface of control and Stim1 cKO incisors. The Stim1 cKO incisors showed a weaker signal with higher gray levels associated with more abraded enamel surfaces. White spots are overcharging artifacts (star). **(D)** Quantitative analysis of relative enamel thickness and BSE gray levels (*p < 0.01).

To assess the effect of loss of STIM1 in enamel, we conducted a detailed examination of the dentition of 4 weeks old male Stim1 cKO mice and compared these to their age-matched control littermates. The teeth shape in the control and cKO mice appeared to be normal. Gross visual inspection of the incisors of Stim1 cKO mice showed an abnormal chalky-white appearance which is often associated with hypomineralization or enamel loss (Figure 2A). Stim1 cKO mice showed greatly reduced enamel at the incisor tips (Figure 2A arrows). The incisor relative enamel thickness (i.e., enamel thickness divided by enamel and dentin thickness) was significantly reduced in Stim1 cKO mice compared to the control (Figure 2B). When imaged using BSE microscopy mode, Stim1 cKO incisors displayed rough enamel surfaces with a lower degree of mineralization and several gray hypomineralized spots (Figure 2C). Indeed, quantitative analysis (*n* = 3 per group) showed that the enamel of the incisors was thinner and less mineralized in Stim1 cKO mice compared to controls (Figure 2D). No noticeable differences were observed in the enamel phenotype of Stim1 cKO between female and male mice (data not shown).

**FIGURE 3:**
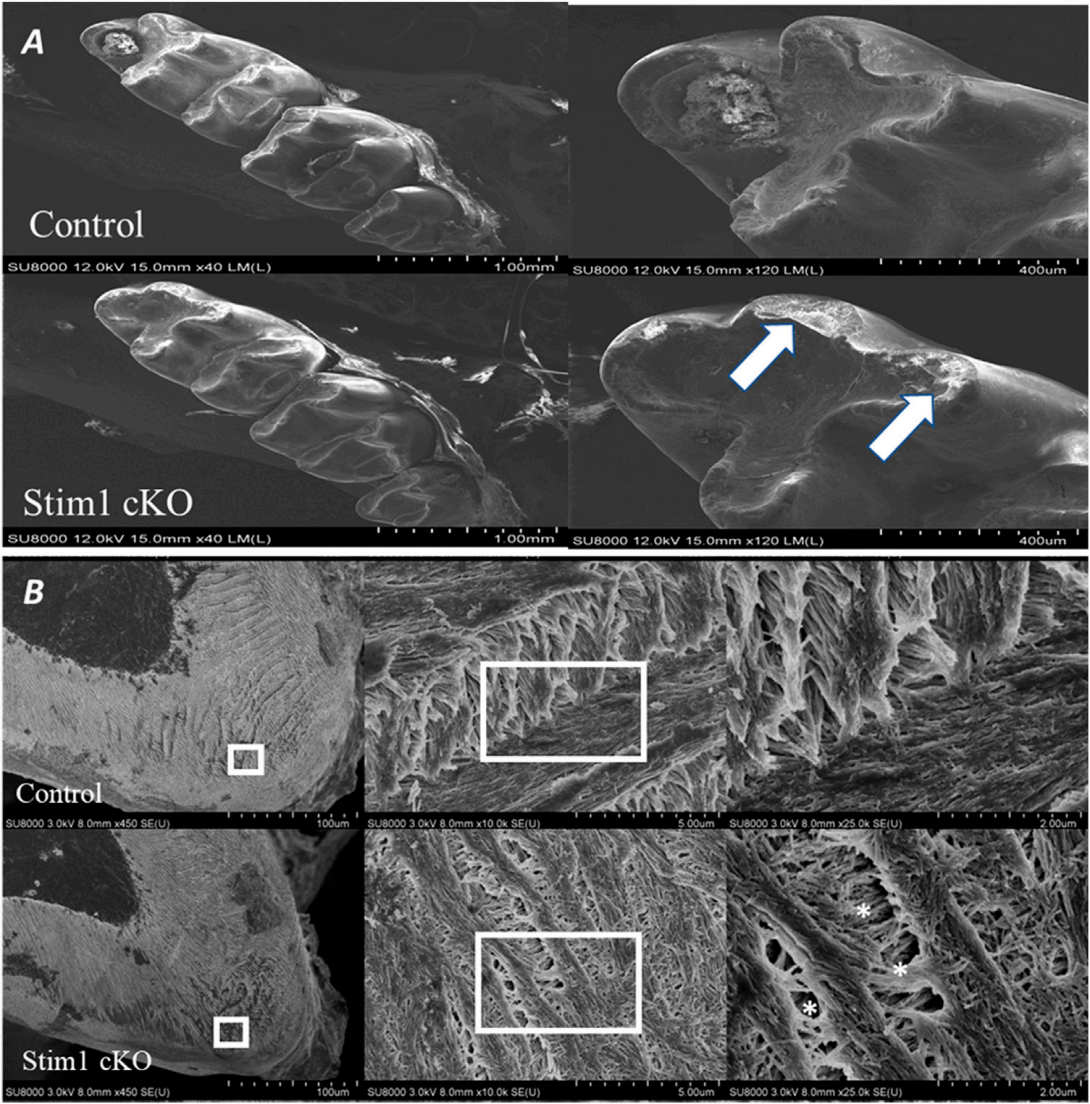
Phenotype analysis of 4-week old control and Stim1 cKO mice molars. **(A)** Representative scanning electron microscope (SEM) images of control and Stim1 cKO mice molars. The number and shape of cusps seem unchanged between Stim1 cKO and control mice. However, Stim1 cKO molars displayed significant attrition on the occlusal surfaces when compared with control. The arrows indicate the attritional defects on the occlusal surfaces of the cusps. **(B)** SEM photographs taken at a sagittal section cut through the mandibular first molar. In the area of the highly resilient wavy gnarled enamel, Stim1 cKO mice showed a less compact condensation of gnarled prisms. Higher magnification of the ground sections revealed that crystallites in the bulk enamel in Stim1 cKO mice were less impacted and enamel rods were thin, disorganized, and more porous (stars) as compared with control mice. The rectangular areas are enlarged in the right panel of each image.

In the molars, the number and shape of cusps were unchanged between Stim1 cKO and control mice. This indicates that no early developmental disruptions in tooth formation occurred as the number and morphology of cusps are established prenatally. However, SEM imaging revealed significant shape changes (attrition) in the cusp tips of Stim1 cKO mice relative to the control (Figure 3A, arrows). In the area of the highly resilient wavy gnarled enamel, Stim1 cKO mice showed a less compact condensation of gnarled prisms which renders the enamel significantly weaker and greatly impairs its ability to withstand occlusal forces (Figure 3B). Indeed, higher magnification of the ground sections revealed that the crystallites in the bulk enamel in Stim1 cKO mice were less impacted. Moreover, enamel rods were thin, disorganized, and more porous compared with control mice (Figure 3B, stars). This disruption of the rod-interrod pattern may be indicative of changes in the depositional pattern of the enamel matrix. The molar enamel thickness was significantly reduced in Stim1 cKO mice compared to the control.

### Stim1 cKO resulted in enamel hypomineralization

**FIGURE 4:**
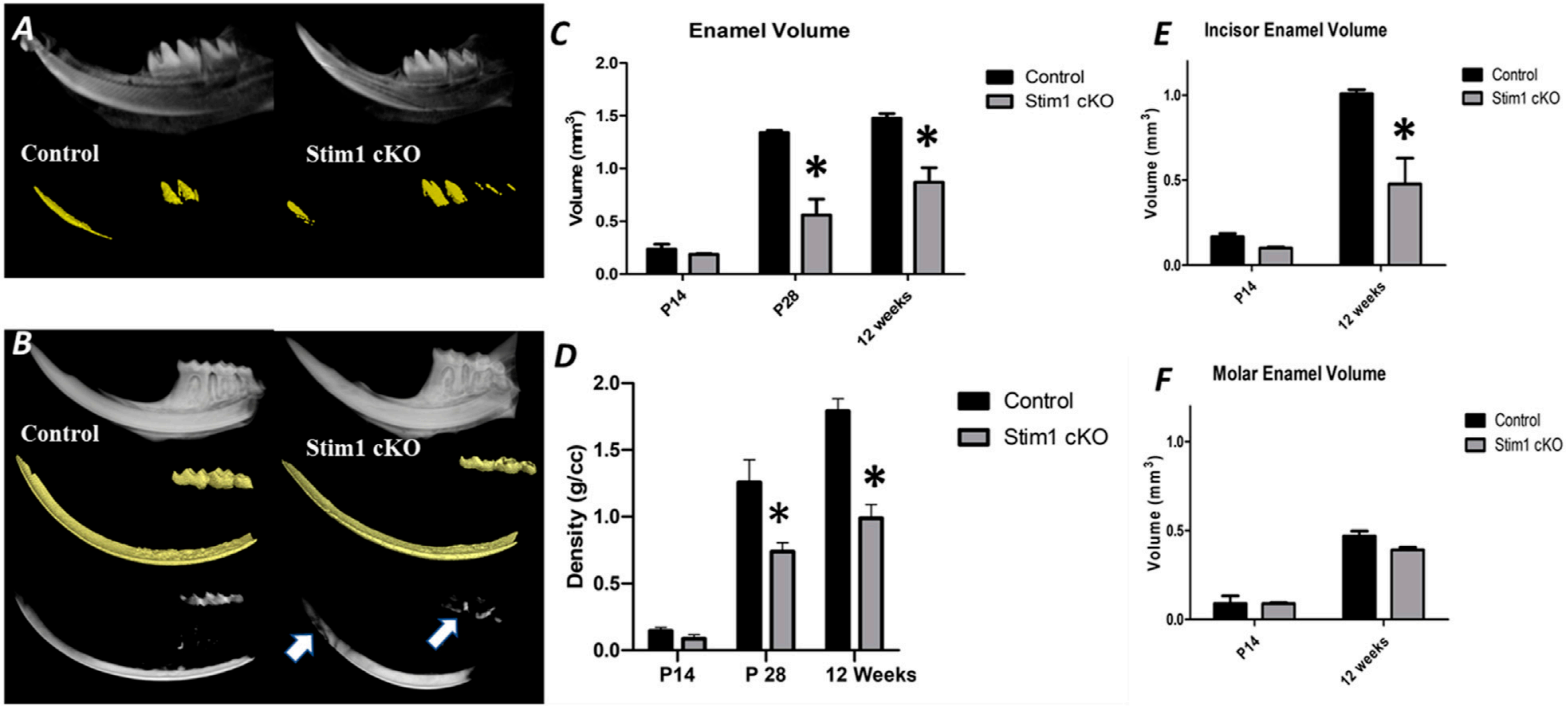
Micro-computed tomography (μCT) analysis of enamel. **(A,B)** A 3D μ CT of the control (left column) and Stim1 cKO (right column) hemimandibles collected from mice at postnatal day 14 (P14) **(A)** and 12 weeks **(B)**. The Stim1 cKO mice showed loss of enamel volume (color-coded gold) and thickness in Stim1 relative to their control littermates in incisors and molars at 12 weeks while no significant differences were noticed at P14. For 12 weeks samples the threshold value for imaging was raised until enamel was the only mineral displayed in the control group. the enamel of the teeth working surfaces (i.e., incisor tips and molar cusps) does not reach the threshold level in the Stim1 cKO mice (B, arrows). Quantitative analysis for enamel volume **(C)** and enamel mineralization **(D)** showed that Stim1 cKO enamel displayed reduced volume and mineral densities that increases with age. The threshold volume of mineralized enamel was reduced in incisors **(E)** more significantly compared to molars **(F)** in both young (P14) and old (12 weeks) mice (mean ± SEM of *n* = 3 mice per group; *p < 0.05).

To investigate the quality and quantity of enamel mineralization, control and Stim1 cKO hemimandibles were imaged by 3D microcomputed tomography (μCT) at 3 time points (2, 4 and 12 weeks). The threshold value for imaging was raised until enamel became the only mineral displayed in the control group (Figures 4A, B). In 4 and 12 weeks old mice, Stim1 cKO mice showed a significantly reduced volume of high-density mineral relative to the control, especially in the mastication surfaces (incisor tips and molar cusps) (Figure 4B, arrows). Quantitative measurements revealed that the enamel volume and mineral density in Stim1 cKO mice were significantly diminished when compared with the enamel of the control mice. These differences became more significantly pronounced as mice aged (Figures 4C, D). Moreover, the threshold volume of mineralized enamel was reduced in incisors more significantly compared to molars in both young (P14) and old (12 weeks) mice (Figures 4E, F).

**FIGURE 5:**
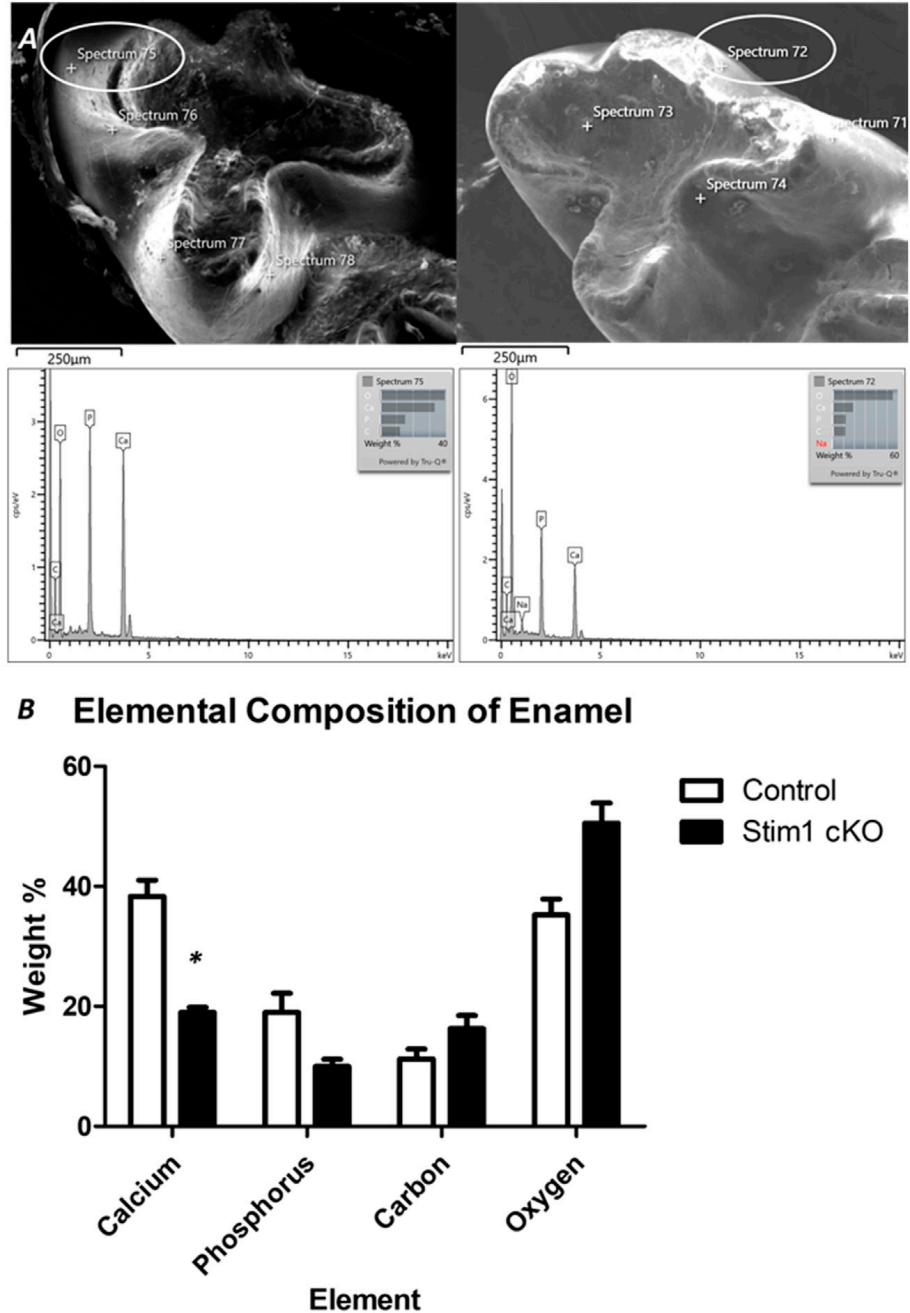
Elemental composition analysis of enamel. **(A)** To assess enamel mineralization, we analyzed changes in elemental composition in enamel using energy dispersive x-ray spectroscopy (EDS). For EDS, 4 beams were generated on the cusp slopes of maxillary first molars that received no processing of the enamel surface in order not to alter its composition. The lower row shows the peaks generated from the circled areas. **(B)** Quantitative analysis of representative average measurements taken by EDS of elemental composition of the enamel of control and Stim1 cKO enamel. Results show that the enamel of Stim1 cKO mice is Ca deficient, with a very significant loss of the Ca amount found in controls (Figure 5B). Other key elements present in mineralized enamel, such as phosphate (P), were also lower in Stim1 cKO mice compare to controls albeit non-significantly. (mean ± SEM of *n* = 3 mice per group; *p < 0.05).

To further assess enamel mineralization, we assessed changes in elemental composition in enamel using energy dispersive x-ray spectroscopy (EDS) (Figure 5A). Results showed that the enamel of Stim1 cKO mice is calcium deficient, with a very significant loss of the calcium amount found in controls (Figure 5B). Other key elements present in mineralized enamel, such as phosphorus, were also lower in Stim1 cKO mice than in controls albeit non-significantly. No significant difference was noted in the Ca/P ratio between the Stim1 cKO mice (1.95 ± 0.25) and control mice (2.08 ± 0.22). However, the Ca/C ratio was significantly lower in Stim1 cKO mice (1.22 ± 0.19) compared to control mice (3.60 ± 0.68). On the other hand, data extrapolated from the previous EDS analysis of the labial surface of the incisors done during our pilot study (Said et al., 2019) showed that *Stim1* deletion resulted in a more significant drop of calcium content and the both the Ca/P and Ca/C were more significantly reduced in Stim1 cKO incisors compared to control (Ca/P; 2.92 ± 0.13 in control incisors and 1.42 ± 0.18 in Stim1 cKO, Ca/C; 5.31 ± 1.48 in control incisors and 1.14 ± 0.07 in Stim1 cKO) (Supplementary Figures S1A, B).

### Altered gene expression in incisors’ ameloblasts of Stim1 cKO mice

**FIGURE 6:**
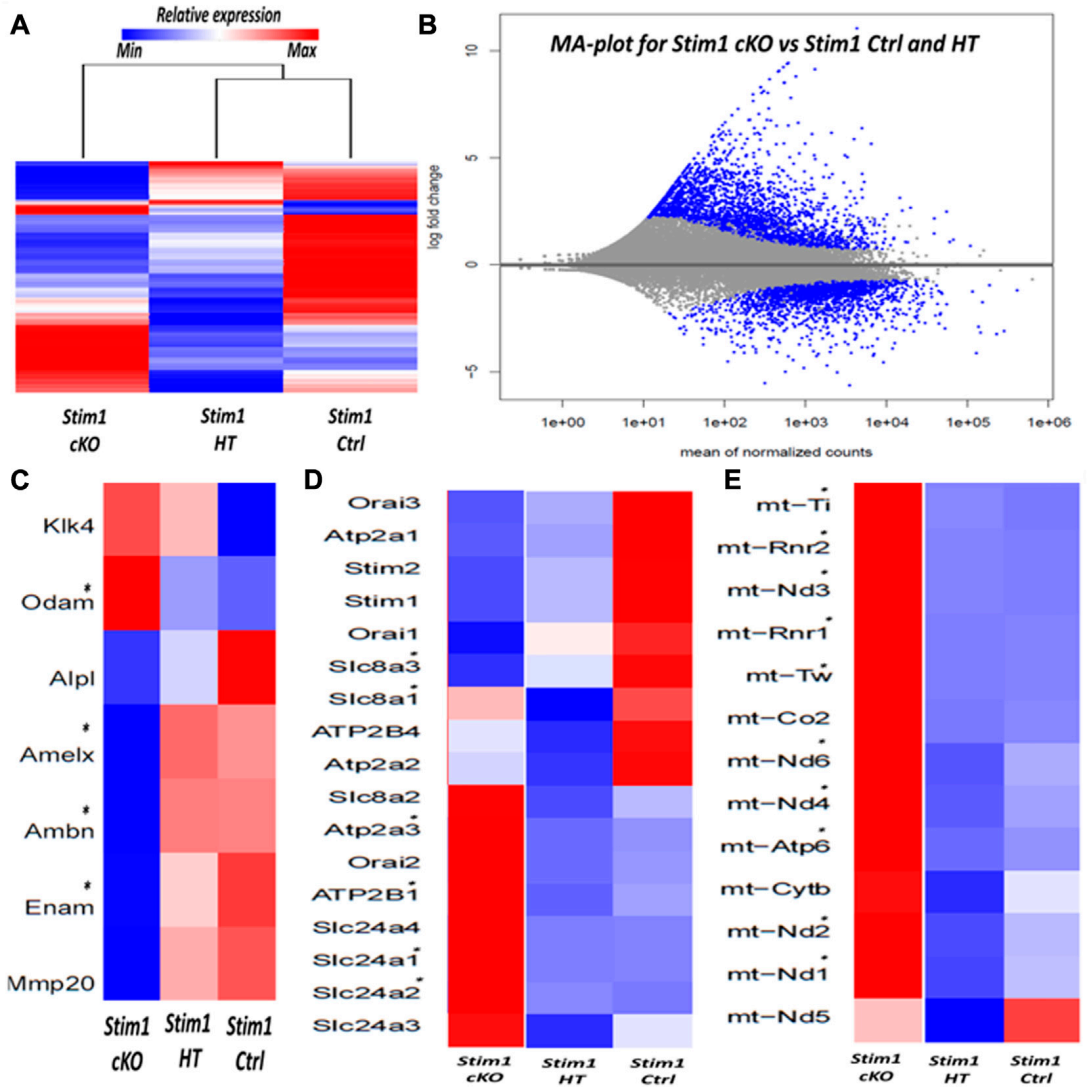
RNA sequencing of enamel organ cells in Stim1 cKO incisors mice highlights changes in enamel proteins and mitochondrial pathways **(A)** Heatmap and dendrogram showing clustering of samples with similar gene expression, the genetic profile of Stim1 HT was closely correlated to Stim1 Ctrl **(B)** MA plot of magnitude of differential gene expression in Stim1 cKO compared to both Stim1 HT and Stim1 Ctrl (shown as the log base 2 of magnitude of mean expression difference on the *y*-axis *versus* the mean of normalized counts on the *x*-axis). The differentially expressed genes (adj. *p* < 0.01, absolute fold change of more than 2) are shown in blue. **(C)** Heatmap based on the expression of enamel genes. Clear differences in expression can be observed in *AmelX, Ambn, Enam and Odam* in Stim1 cKO compared to both Stim1 HT and Stim1 Ctrl. No clear differences were found between the Stim1 HT and Stim1 Ctrl in the expression of the main enamel genes **(D)** Heatmap based on the expression of genes involved in calcium transport. Several differences in expression can be observed in genes encoding the calcium pumps SERCA3, PMCA1 in addition to the calcium exchangers NCKX1 and NCKX2 in Stim1 cKO compared to both Stim1 HT and Stim1 Ctrl. NCKX4 was also upregulated in Stim1 cKO mice with an adjusted *p*-value that is near significance (adj. *p* < 0.05) **(E)** A heatmap showing the expression levels of several mitochondrial RNA’s. Of interest, expression of several NADH dehydrogenase and cytochrome oxidase subunits were upregulated in Stim1 cKO amelobalsts. No clear differences were found between the Stim1 HT and Stim1 in the expressions of most of these mitochondrial subunits (*adjusted *p* < 0.01).

To analyze the effects of SOCE deficiency in ameloblasts more comprehensively, we extracted RNA from the entire ameloblast populations isolated from the whole incisor enamel organ (EO) of P28 old mice and analyzed global gene expression by RNA sequencing (RNAseq). The ameloblasts’ RNA was extracted from 3 samples (*n* = 1 per genotype); 1 Stim1 cKO sample with complete deletion of *Stim1*, 1 Stim1 HT sample with heterozygous deletion of one *Stim1* allele and 1 Stim1 Ctrl with no *Stim1* deletion as it was Cre negative. Interestingly, Stim1 heterozygous knock-out mice did not show a clear loss-of-function enamel phenotype which may indicate that the *Stim1* gene is relatively haplosufficient during amelogenesis. Moreover, hierarchical clustering of the samples showed that the Stim1 HT and Stim1 Ctrl had a relatively similar gene expression profiles (Figure 6A). The three samples showed very low read counts of vimentin when compared to the much higher read counts of *AmelX* and *Ambn* transcripts, which indicates a minimal degree of mesenchymal contamination. We identified a total of 2277 differentially expressed genes in Stim1 cKO compared to the grouped Stim1 HT and Ctrl cells, of which 1264 were significantly upregulated and 1014 genes were significantly downregulated (adjusted *p*-value <0.01, absolute fold change >2) (Figure 6B) (Supplementary Datasheet S1).

The exploratory RNAseq data indicate that *Stim1* deficient mice showed considerably lower average values in the expression of the enamel genes *AmelX, Enam and Ambn* and higher average values in the expression of Odontogenic ameloblast-associated protein (*Odam*) compared to both Stim1 HT and Ctrl cells (Figure 6C). Moreover, loss of *Stim1* appears to cause additional changes in the expression of several genes that encode calcium pumps and exchangers that are thought to be involved in transcellular calcium transport during amelogenesis. Indeed, Stim1 cKO ameloblasts showed higher expression levels of the SERCA3 pump (encoded by the gene *Atp2a3*) and the Plasma membrane Ca2+ ATPase 1 (PMCA1 pumps) (encoded by the *Atp2b1* gene). Furthermore, the expression levels of the Na+/Ca2+-K+ exchangers 1 & 2 (NCKX1 & 2; encoded by the genes *Slc24a1 & 2,* respectively) were also upregulated in Stim1 cKO ameloblasts (Figure 6D). NCKX4 was also upregulated in Stim1 cKO mice with an adjusted *p*-value that is near significance (adj. *p* < 0.05) when compared to both Stim1 HT and Stim1 Ctrl. Panther pathway analysis showed that ATP synthesis is the most enriched pathway in EO cells of Stim1 cKO mice (Supplementary Table S2). Indeed, the expression levels of several subunits of the respiratory chains’ mitochondrial proteins NADH dehydrogenase and cytochrome oxidase were upregulated in Stim1 cKO ameloblasts compared to both Stim1 HT and Ctrl cells (Figure 6E).

**FIGURE 7:**
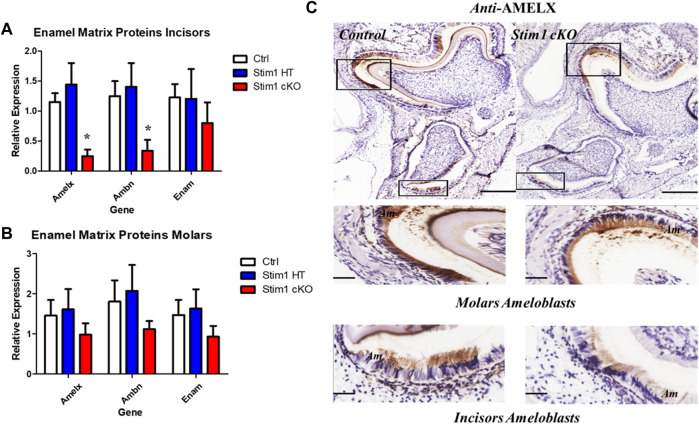
Enamel matrix genes and proteins expression analysis of Stim1 cKO mice. Gene expression analysis in incisors **(A)** and molars **(B)** ameloblasts from control and Stim1 cKO mice (mean ± SEM, *n* = 3∼4 mice per genotype; *p < 0.05 compared to Ctrl). Our analysis showed that the expression levels of the genes encoding the enamel matrix proteins AmelX and Ambn were upregulated in Stim1 cKO incisors ameloblasts compared to control. However, no significant changes were observed in the molars ameloblasts albeit having lower RNA levels of AmelX, Ambn and Enam. No clear differences were found between the Stim1 HT and Stim1 Ctrl in the expression of the main enamel genes. **(C)** Immunostaining of the mandibular molar and incisor tooth buds showed reduced protein levels of Amelogenin in Stim1 cKO as compared with age-matched controls. The change was more pronounced in incisors’ ameloblasts compared to molars. The rectangular areas are enlarged in the lower panels for each image. Scale bars: **(C)** 250 µm in the upper low mag panels and 50 µm in the lower high mag panels.

To further explore these changes and attain statistical significance, we performed RT-qPCR analysis on ameloblasts isolated from the molars and incisors of mice aged between P10 to P12 (*n* = 3 to 4 per genotype) to asses changes in the expression levels of several genes of interest. Our analysis showed that the expression levels of the genes encoding the enamel matrix proteins *AmelX* and *Ambn* were upregulated in Stim1 cKO incisors ameloblasts compared to control (Figure 7A). However, no significant changes were observed in the molars ameloblasts albeit having lower RNA levels of *Amelx, Ambn and Enam* (Figure 7B). No clear differences were found between the Stim1 HT and Stim1 Ctrl in the expression of the main enamel genes which may explain the lack of phenotype in Stim1 HT mice. Furthermore, immunostaining for the AMELX protein was performed to supplement the qPCR results. Immunostaining showed a weaker signal AMELX in Stim1 cKO compared to their sex- and age-matched controls but the change was more pronounced in incisors’ ameloblasts compared to molars (Figure 7C). We also performed immunostaing of enamel proteases MMP20 and KLK4 which also showed a weaker signal of both proteins in Stim1 cKO compared to their sex- and age-matched controls albeit the change was less clearly observed in KLK4 (Supplementary Figure S2).

**FIGURE 8:**
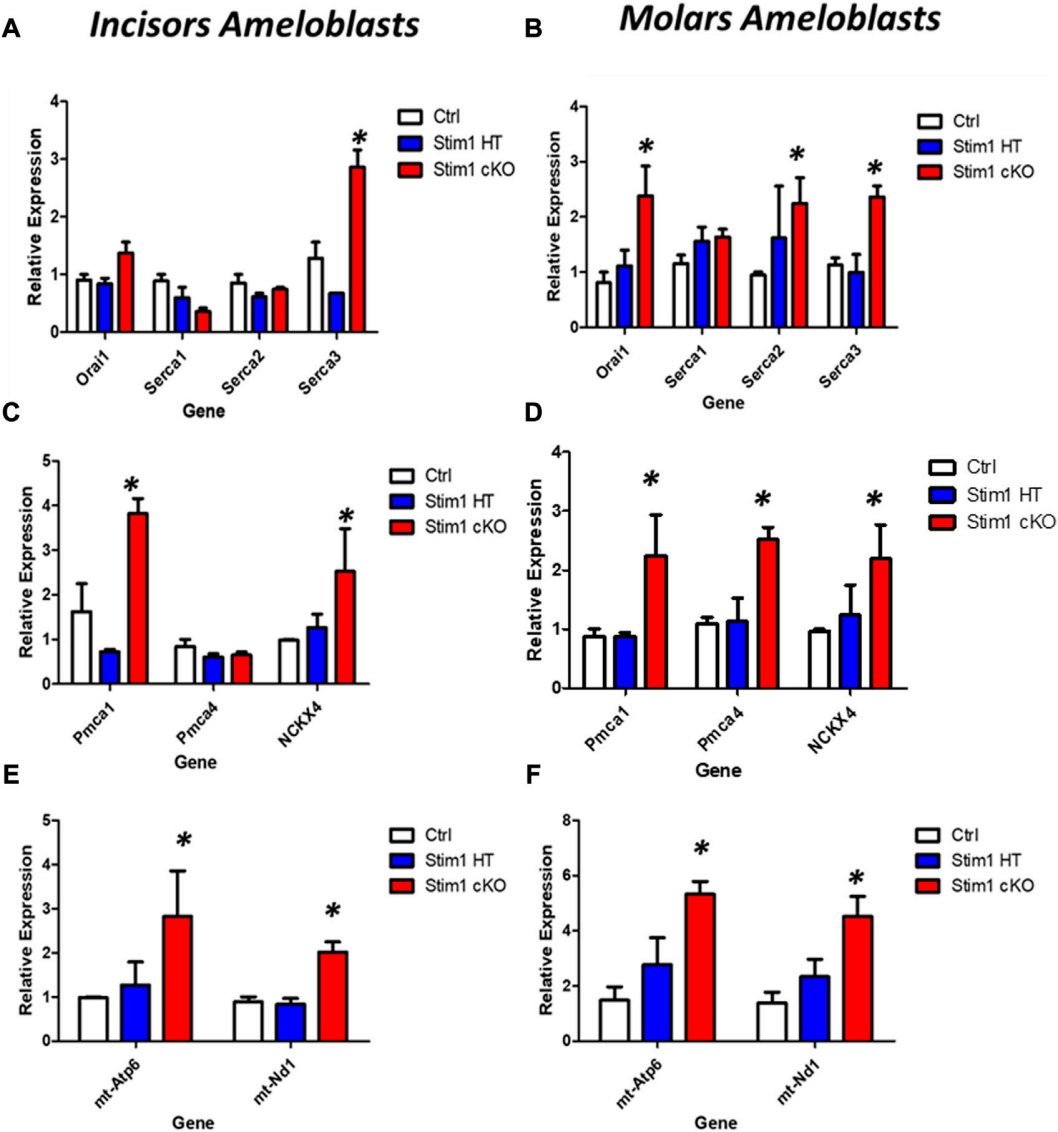
Calcium transport and mitochondrial gene expression analysis of Stim1 cKO mice. **(A–D)** Expression analysis for genes involved in calcium transport in molars and incisors ameloblasts collected from control and Stim1 cKO mice. Our RT-qPCR analysis showed that Stim1 deletion has resulted in significant upregulation of several genes encoding proteins that are essential for SOCE and transcellular calcium transport during amelogenesis. These changes appear to be more pronounced in molars’ ameloblasts as the 7 genes we investigated molars Stim1 cKO ameloblasts had a significantly higher expression of 6 genes (SECA2, SERCA3, NCKX4, PMCA1 and PMCA4) compared to control (genes encoding ORAI1, SERCA1,2,3, PMCA1,4 and NCKX 4) while Stim1 cKO incisors ameloblasts displayed a significantly higher expression of three genes involved in calcium transport (SERCA3, NCKX4 and PMCA1) compared to control. Similar to the genes encoding the enamel matrix proteins, no clear differences were found between the Stim1 HT and Stim1 Ctrl in the expression of the seven genes. **(E,F)** Expression analysis for the mitochondrial genes (mt-Atp6 and mt-Nd1) in molars and incisors ameloblasts collected from control and Stim1 cKO mice. The expression levels of both mitochondrial genes were significantly upregulated in Stim1 cKO ameloblasts compared to controls in both incisor and molar ameloblasts. However, the degree of upregulation was more significant in Molars’ ameloblasts. No clear differences were found between the Stim1 HT and Stim1 Ctrl in the expressions of these mitochondrial genes (mean ± SEM, *n* = 3∼4 mice per genotype; *p < 0.05 compared to Ctrl).

As for genes involved in calcium transport, our RT-qPCR analysis showed that *Stim1* deletion has resulted in significant upregulation of several genes encoding proteins that are essential for SOCE and transcellular calcium transport during amelogenesis. However, these changes appear to be more pronounced in molars’ ameloblasts. Indeed, of the 7 genes we investigated (genes encoding ORAI1, SERCA1,2,3, PMCA1,4 and NCKX 4), our results showed that Stim1 cKO incisors ameloblasts displayed a significantly higher expression of three genes involved in calcium transport (SERCA3, NCKX4 and PMCA1) compared to control while molars Stim1 cKO ameloblasts had a significantly higher expression of 6 genes (SECA2, SERCA3, NCKX4, PMCA1 and PMCA4) compared to control (Figures 8A–D). Similar to the genes encoding the enamel matrix proteins, no clear differences were found between the Stim1 HT and Stim1 Ctrl in the expression of the aforementioned genes. Finally, the expression levels of the mitochondrial RNAs *mt-ND1* and *mt-Atp6* were significantly upregulated in Stim1 cKO ameloblasts compared to controls in both incisor and molar ameloblasts (Figures 8E, F). However, the degree of upregulation was more significant in Molars’ ameloblasts. No clear differences were found between the Stim1 HT and Stim1 Ctrl in the expressions of these mitochondrial genes which may further explain the lack of a clear enamel phenotype in Stim1 HT mice.

## Discussion

The data presented here demonstrate that Stim1 cKO mice may serve as a valuable model for assessing the effects of Ca2+ deficiency in enamel. Abolishing SOCE resulted in the development of a clearly defective enamel phenotype and altered ameloblast function. In particular, these findings implicate SOCE channels as essential players in the mineralization process of dental enamel. In addition, our data also suggest that SOCE-mediated calcium entry influences several ameloblasts’ cellular functions including enamel secretion and oxidative phosphorylation. Furthermore, our data suggest that the effects of *Stim1* deletion differs considerably between incisors and molars. Indeed, our results suggest that SOCE invalidation in incisors’ ameloblasts has resulted in a higher degree of enamel hypomineralization associated with reduced enamel matrix proteins synthesis and lower mitochondrial activity and biogenesis compared to molars. Finally, our results showed that partial heterozygous deletion of *Stim1* does not appear to cause significant changes in the enamel phenotype and ameloblasts genotype.

Consistent with abnormal amelogenesis, our morphometric analysis of Stim1 cKO dentition revealed that the enamel of *Stim1* deficient mice was thinner and presented with impaired structural integrity compared to controls. The surface enamel of Stim1 cKO mice produced weaker BSE-SEM signals relative to controls, which is highly consistent with decreased mineralization. This hypomineralization was further confirmed by micro-computed topography and EDS elemental composition analysis of enamel, showing decreased mineral density and diminished calcium content, respectively. Our micro-CT analysis showed that enamel defects were more pronounced in older mutant mice compared to younger ones and the threshold volume of mineralized enamel was reduced in incisors more significantly compared to molars in both young (P14) and old (12 weeks) mice. We hypothesize that Stim1 cKO ameloblasts did form enamel during the early stages of amelogenesis, however, the enamel was mineral deficient and mechanically weaker resulting in a more rapid rate of wear and attrition with time, ensuing a stronger enamel phenotype with age. The reduced mineral content observed early in the incisor enamel rendered it more susceptible to attrition compared to molars. No significant difference was noted in the Ca/P ratio between the Stim1 cKO mice and control mice molars as both elements were reduced after *Stim1* depletion. However, the Ca/C ratio was significantly lower in Stim1 cKO mice compared to control mice which may indicate that Stim1 cKO enamel matrix has a higher organic content compared to controls. On the other hand, EDS analysis of the labial surface of the incisors showed that *Stim1* deletion resulted in a more significant drop of calcium content and the both the Ca/P and Ca/C were more significantly reduced in Stim1 cKO incisors compared to control which further suggests a higher degree of enamel hypomineralization in incisors compared to molars. Finally, Stim1 cKO enamel showed an abnormal prismatic pattern and the structural integrity of the enamel of Stim1 cKO mice appears to be significantly weaker. Generally, the enamel phenotype observed in these mice resembles the hypomineralized amelogenesis imperfecta observed in ectodermal dysplasia patients with impaired SOCE (Lacruz and Feske, 2015).

As has been described in previous reports (Lacruz et al., 2011; Lacruz et al., 2012; Nurbaeva et al., 2015a; Nurbaeva et al., 2015b; Eckstein and Lacruz, 2018), we found that expression of *Stim1* is higher in the maturation stage ameloblasts as compared to those in the secretory stage in both incisor and molars ameloblasts. Moreover, the ablation of *Stim1* did not affect the expression levels of *Stim2*. It has been previously shown that while the *Stim2* mRNA expression was as abundant as that of *Stim1* in maturation ameloblasts, the STIM1 protein levels are higher than those of STIM2 (Lacruz et al., 2012). Thus, the fact that our Stim1 cKO mice exhibited dramatically suppressed enamel mineralization despite still having a functional *Stim2* gene may be attributed to the reported difference in the production levels of STIM1 and STIM2, which indicates that STIM1 is the main ER calcium sensor in enamel. Indeed, other models with Stim2 cKO did not cause any apparent enamel phenotypes (Furukawa et al., 2017; Eckstein and Lacruz, 2018). which further suggests that STIM1 plays a more significant role in regulating Ca+ during amelogenesis.

In addition to its role in the transcellular Ca2+ transport across the ameloblasts during enamel mineralization, STIM1 and SOCE-mediated calcium entry may play an important role in regulating enamel gene expression during the secretory stage of amelogenesis. Indeed, it has been established that *Stim1* mRNA and protein are expressed in rodent teeth throughout the secretory stage of amelogenesis (Lacruz et al., 2012; Nurbaeva et al., 2015a; Chen et al., 2018). In fact, Chen et al., 2018 have shown that STIM1 protein can be clearly detected in murine ameloblasts, odontoblasts and craniofacial osteoblasts as early as postnatal day 3. SOCE-mediated signalling in ameloblasts is rendered more intriguing by the well-known role of calcium as a second messenger with a broad signalling profile. Our gene expression analysis of incisors’ ameloblasts strongly suggests that SOCE-mediated signalling may affect the expression of the genes encoding main secretory enamel proteins as Stim1 cKO ameloblasts showed a considerably lower expression *AmelX, Ambn* compared to control. On the other hand, RT-qPCR analysis showed lower but non-significant differences in the average expression levels of *AmelX and Ambn* in molar ameloblasts of *Stim1* deficient mice and immunostaining showed a weaker signal AMELX in Stim1 cKO compared to their sex- and age-matched controls, but the change was more pronounced in incisors’ ameloblasts compared to molars. These inconsistent and sometimes contradictory effects of SOCE ablation on enamel matrix proteins gene expression have been reflected in the literature. For example, Nurbeava et al. (2015b) showed that the expression of these enamel genes was upregulated by an increase in cytosolic [Ca2+] mediated by stimulation of SOCE with thapsigargin in both the enamel cell line LS8 and in primary EO cells. Subsequent inhibition of SOCE in LS8 cells has reversed this effect which suggest that SOCE invalidation downregulate the enamel matrix proteins expression similar to what we observed in our incisors (Nurbaeva et al., 2015b). On the other hand, RNAseq data of Stim^1/2K14cre^ cKO mice showed lower but non-significant average values in the expression of *AmelX* as well as *Enam* similar to what we observed in our molars ameloblasts (Eckstein et al., 2017). Another analysis of Stim^1/2K14cre^ cKO molars ameloblasts by Furukawa et al., 2017 showed no significant differences in the expression levels of *AmelX* and *Ambn* (Furukawa et al., 2017)*.* Finally, a more recent study by Eckstein et al., 2019 showed that the expression of *AmelX* was slightly increased while *Enam* was significantly increased in EO cells of Orai1^K14^ cKO mice and expression of both genes was significantly decreased in enamel cells of Orai2 null mice with no differences detected in *Ambn* in either Orai1^K14^ cKO or Orai2 null mice. Based on all of the above, it appears that SOCE mediated calcium signalling may play a minor yet heterogenous role during enamel secretion in addition to its well-established role late in enamel mineralization and maturation. However, it remains unclear how the different components of SOCE affects the expression of the different main enamel matrix proteins and a more specific analysis like single cell genomics are still needed to explain this apparent heterogeneity of the results.

On the protein level, we also analyzed AMELX, MMP20 and KLK4 protein expression levels and found weaker signals in the enamel of Stim1 cKO mice thus further suggesting the overall downregulation of key ameloblast proteins in Stim1 cKO. AMELX is the most abundant enamel matrix protein (90%) and is essential for amelogenesis (Shin et al., 2020). Amelogenin-deficient mice are known to display a severe AI phenotype (Simmer et al., 2021). MMP20 and KLK4 are the most crucial proteases for the proper processing, degradation, and uptake of enamel matrix proteins by ameloblasts during enamel maturation and mineralization (Furukawa et al., 2017). In mice lacking KLK4, a defective rod-interod arrangement is observed in the first formed inner enamel near the dentino-enamel junction (Goldberg et al., 2014). Thinner and structurally abnormal incisor enamel is observed in MMP20 KO mice (Simmer et al., 2021). Generally, *AmelX, Mmp20 and Klk4* KO mice all show different degrees of enamel AI phenotype including hypoplasia, hypomineralization, and hypomaturation (Wright et al., 2008; Simmer et al., 2010). This is consistent with our findings of AI phenotype observed in our Stim1 cKO where AMELX, MMP20 and KLK4 proteins production appears to be downregulated.

Our gene expression analysis showed that *Stim1* ablation has resulted in significant upregulation of genes encoding the ATP-dependent calcium pumps SERCA3 and PMCA1 in addition to the calcium exchanger NCKX4 in incisors ameloblasts. The aforementioned 3 genes were also significantly upregulated in molars Stim1 cKO ameloblasts in addition to the 2 other isoforms of calcium pumps (SERCA2 and PMCA4) and the SOCE component ORAI1. SERCAs transport cytosolic Ca2+ into the ER lumen to replenish the intracellular stores, whereas PMCAs extrude cytosolic Ca2+ out of the cell (Bronckers, 2017). The three isoforms of SERCA pumps have been shown to be expressed in rodent ameloblast both *in vitro* and *in vivo* (Nurbaeva et al., 2015a; Nurbaeva et al., 2015b). Several reports have suggested that PMCAs may play an important role in calcium transport during amelogenesis as they were strongly expressed during both the secretory and maturation stages of amelogenesis with PMCA1 and 4 being the dominant isoforms (Borke et al., 1995; Zaki et al., 1996; Bomfim et al., 2023). NCKXs are believed to be involved in Ca2+ clearance from ameloblasts as all NCKX isoforms are expressed by rodent ameloblasts (Nurbaeva et al., 2017; Paine et al., 2020). The NCKX4 in particular is thought to be critical for enamel maturation as it is heavily expressed in maturation-stage ameloblasts (Bronckers, 2017). This crucial role of NCKX4 in enamel mineralization has been further elucidated by studies showing that mutations in NCKX4 in humans and mice result in abnormal hypomineralized enamel similar to the enamel observed in patients with SOCE mutation (Parry et al., 2013; Khan et al., 2020). It is possible that the increased expression of SERCA and PMCA pumps observed here in Stim1 cKO enamel cells is needed to compensate for the lower cytosolic concentration and maintain some level of calcium transport during enamel maturation. The higher degree of upregulation of these pumps in addition to upregulation of ORAI1 in molars compared to incisors may explain the lower degree of hypomineralization observed in Stim1 cKO molar enamel compared to incisors. An increased expression of SERCA2 has been reported in ORAI1-deficient cells compared to control cells to enhance the ER calcium replenishing (Eckstein et al., 2019). Overexpression of STIM1 was shown to attenuates PMCA-mediated Ca2+clearance in T-lymphocytes cell lines (Ritchie et al., 2012) However, no changes in either the SERCA or PMCA pumps were reported in Stim^1/2K14cre^ cKO ameloblasts. As for NCKX4, contradictory results have been reported on the effect of SOCE on this apical ion transporter. Eckstein et al. (2017) showed that NCKX4 localization was dysregulated in Stim^1/2K14cre^ and attributed it to disrupted ameloblasts cytoskeletal assembly of ruffled border (Eckstein et al., 2017). On the other hand, Furukawa et al. (2017) found no obvious differences in the expression and localization NCKX4 in Stim1/2^K14Cre^ ameloblasts compared to the control. Generally, and similar to the effects on enamel matrix proteins, further studies are still needed to fully evaluate the exact state of calcium transport after SOCE dysregulation.

Among the top functions identified in the pathway analysis of the differentially expressed genes, ATP synthesis was the most prominent. Indeed, our gene expression data showed that SOCE ablation has resulted a very significant upregulation of mitochondrial gene expression which suggests an increased rate of mitochondrial biogenesis, ATP production and reactive oxygen species (ROS) production in ameloblasts. The degree of mitochondrial genes upregulation was more significant in molars ameloblasts compared to incisors. We postulate that a higher mitochondrial metabolic activity may be needed to compensate for the increased ATP demand by the upregulated ATP dependent calcium pumps (i.e., SERCAs and PMCAs) observed in Stim1 cKO ameloblasts. The higher rate of mitochondrial genes expression upregulation observed in molars may correspond to the previously noted higher rates of calcium pumps over-expression in molars ameloblasts which may further explain the lower degree of hypomineralization observed in Stim1 cKO molar enamel compared to incisors. The links between SOCE and mitochondrial homeostasis are well documented in the literature (Hawkins et al., 2010; Henke et al., 2012; Srikanth and Gwack, 2012; Deak et al., 2014). For example, Henke et al., 2012 showed that STIM1 and ORAI1 deficiency in murine fibroblasts rendered the cells more susceptible to oxidative stress, which was later rescued by STIM1 and ORAI1 overexpression (Henke et al., 2012). Moreover, they showed that Stim1 knock-out mitochondria are tubular and are metabolically more active, resulting in constitutive oxidative stress (Henke et al., 2012). The upregulation of the electron transport chain components in our Stim1 cKO EO cells suggests that *Stim1* deletion may eventually lead to a similar increased metabolic activity and increased oxidative stress and reactive oxygen species (ROS) production in ameloblasts. In the context of amelogenesis, SOCE abrogated ameloblasts of Stim1/2^K14cre^ cKO mice generated by Eckstein et al. (2017) showed abnormal mitochondrial morphology and a significant increase in ROS levels compared with control cells as the oxidative stress response and glutathione metabolism pathways were the most enriched in EO cells from Stim1/2^K14cre^ cKO mice (Eckstein et al., 2017). Furukawa et al. (2017) also suggested that SOCE mediated mitochondrial dysfunction maybe one of the main underlying mechanisms involved in the development of the AI phenotype observed in their Sitm1/2 cKO mice as well (Furukawa et al., 2017). Furthermore, ORAI1-deficient LS8 enamel cells showed a higher oxygen consumption and ATP production rate compared to ORAI2-and ORAI3-deficient or control cells (Eckstein et al., 2019). Collectively, all these data strongly suggest an abnormal mitochondrial function in *Stim1*-deficient ameloblasts and highlight the relevance of mitochondrial apparatus in enamel forming cells, an area that still needs more research.

Generally, our data suggest that SOCE dysregulation exerts a differential effect on the enamel phenotype and ameloblasts genotype between incisors and molars. Indeed, our results suggest that SOCE invalidation in incisors’ ameloblasts has resulted in a higher degree of enamel hypomineralization associated with reduced enamel matrix proteins and mitochondrial biogenesis compared to molars. These differences may be attributed to the several structural and molecular differences observed between the incisor and molar enamel. Indeed, several differences of enamel phenotype were also observed between incisor and molar from other knock-out (KO) mice depleted for genes necessary for enamel secretion and maturation (Goldberg et al., 2014). For example, The Na + -independent anion exchanger 2 (Ae2) plays a role in pH modulation during the maturation phase of amelogenesis (Goldberg et al., 2014). In Ae2 KO mice, a more pronounced decrease in mineral content and the presence of a high organic matrix are observed in the incisors compared to molars(Lyaruu et al., 2008). Furthermore, αvβ 6 integrins deficient mice also show a more severely defective enamel phenotype in the incisor compared to molars (Mohazab et al., 2013). The differences in the enamel phenotype and genetic profiles between molars’ and incisors’ ameloblasts of Stim1 cKO mice need to be further tested in the future. A more comprehensive comparison between molars and incisors in Stim1 cKO mice is still required to fully confirm the discrepancies between the two teeth observed here. Finally, our results suggest that heterozygous partial deletion of *Stim1* does not appear to cause significant changes in the ameloblasts genetic profile and no significant changes were observed in the expression levels of the 12 genes we analyzed here between Stim1 HT and control mice.

In conclusion, this study reveals that *Stim1* plays a critical role in enamel maturation and mineralization as its abrogation has resulted in the development of a hypomineralized enamel phenotype that is more severe in incisors. In addition to its role of regulating calcium transport during enamel mineralization, gene expression analysis showed that several genes were differentially expressed in Stim1 cKO incisor ameloblasts, suggesting that SOCE-mediated signalling may play critical roles in amelogenesis. Indeed, our data suggest that *Stim1* may regulate enamel matrix protein expression and mitochondrial function during amelogenesis. However, it must be noted that *Stim1* disruption appears to cause a diverse effects of the abovementioned pathways that considerably vary between molars and incisors ameloblasts. The main limitation of this study is the lack of a sufficient number of biological replicates in the RNAseq analysis as the global gene expression analysis presented here is only intended to serve as an exploratory pilot investigation. Additional gene and protein expression studies are still needed to comprehend the full spectrum of *Stim1* downstream targets in ameloblasts.

## Data Availability

The datasets presented in this study can be found in online repositories. The names of the repository/repositories and accession number(s) can be found in the article/Supplementary Material.
