# Supplementary material for: Deciphering the functions of Stromal Interaction Molecule-1 in amelogenesis using AmelX-iCre mice
Source: Front Physiol. 2023 Mar 1;14:1100714. doi: 10.3389/fphys.2023.1100714 (PMC10014868; doi:10.3389/fphys.2023.1100714)
Supplement: Supplementary file 1 [file Table1.DOCX]

**Supplemental Table 1. Primer sequences used for quantitative PCR**

| **Gene** | **5’- Sequence -3’** |
| --- | --- |
| ***b-actin*** | **Forward AAGTACCCCATTGAACACGG**  **Reverse ATCACAATGCCAGTGGTACG** |
| ***Amelx*** | **Forward TACCACCTCATCCTGGGAGC**  **Reverse CTGTTGAGACAGCACAGGGA** |
| ***Abn*** | **Forward CAGAAGGCTCTCCACTGCAA**  **Reverse CCCCAAGGGTGTGGTAACAT** |
| ***Stim1*** | **Forward TGAAGAGTCTACCGAAGCAGA**  **Reverse AGGTGCTATGTTTCACTGTTG** |
| ***Stim2*** | **Forward CGAAGTGGACGAGAGTGATGA**  **Reverse GGAGTGTTGTTCCCTTCACATT** |
| ***Enam*** | **Forward TGCAGAAATCCGACTTCTCCT**  **Reverse CATCTGGAATGGCATGGCA** |
| ***Orai 1*** | **Forward GCTCTGCTGGGTGAAGTTCT**  **Reverse AAGTGAACGGCAAAGACGAT** |
| ***PMCA1*** | **Forward TGGCAAACAACTCAGTTGCATATAGTGG**  **Reverse TCCTGTTCAATTCGACTCTGCAAGCCTCG** |
| ***PMCA4*** | **Forward AAGAAGATGATGAAGGACAACAAC**  **Reverse GTTGCGTACCATATTGTCTCGGTC** |
| ***Serca 1*** | **Forward TGTTTGTCCTATTTCGGGGTG**  **Reverse AATCCGCACAAGCAGGTCTTC** |
| ***Serca 2*** | **Forward GAGAACGCTCACACAAAGACC**  **Reverse CAATTCGTTGGAGCCCCAT** |
| ***Serca 3*** | **Forward CGTCGCTTCTCGGTGACAG**  **Reverse AAGAGGTCCTCAAACTGCTCC** |
| ***NCKX4*** | **Forward GTCGCTCTCACTGTCCTTG**  **Reverse AAGCACAGGAAGACAGCATAG** |
| ***MT-ATP6*** | **Forward TAGCCATACACAACACTAAAGGACGA**  **Reverse GGGCATTTTTAATCTTAGAGCGAAA** |
| ***MT-ND1*** | **Forward CCACCTCTAGCCTAGCCGTTTA**  **Reverse GGGTCATGATGGCAGGAGTAAT** |
