# Supplementary figures and images for: Deciphering the functions of Stromal Interaction Molecule-1 in amelogenesis using AmelX-iCre mice
Source: Front Physiol. 2023 Mar 1;14:1100714. doi: 10.3389/fphys.2023.1100714 (PMC10014868; doi:10.3389/fphys.2023.1100714)

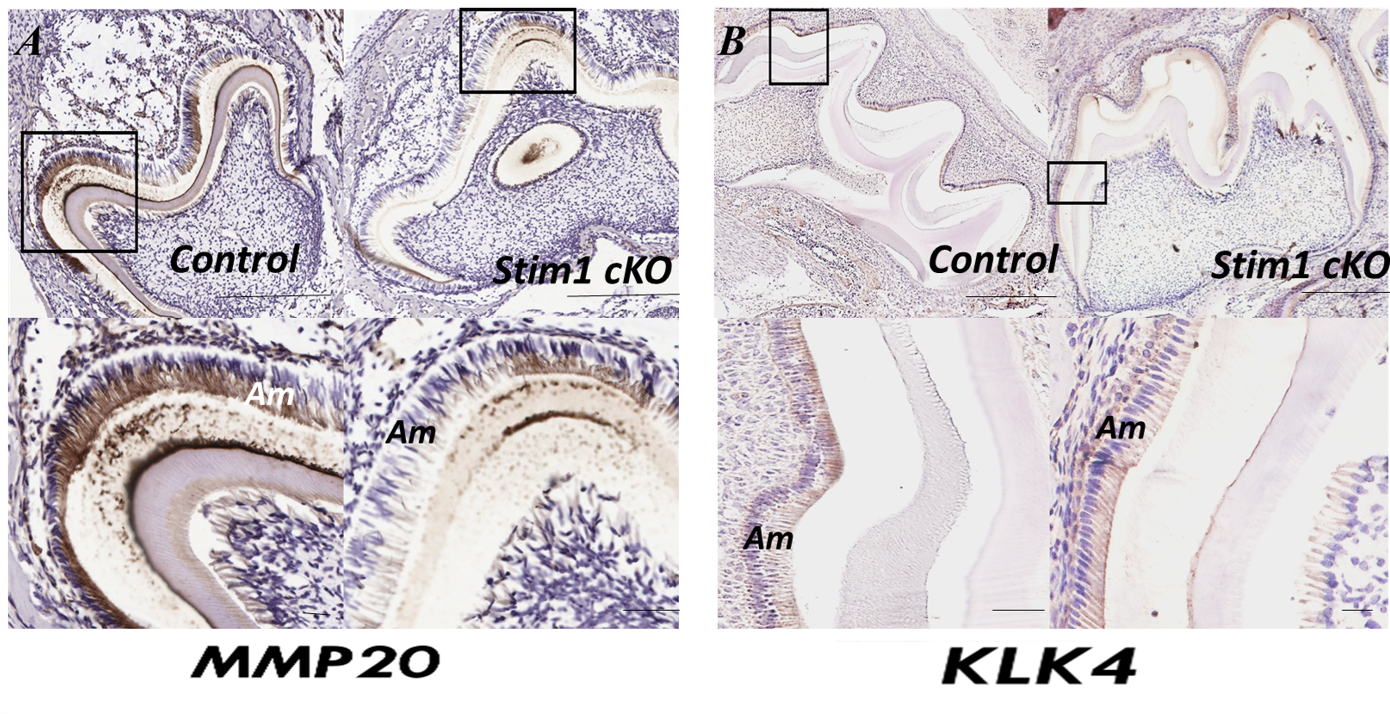

Supplement: Supplementary file 2 [file Image2.TIF]

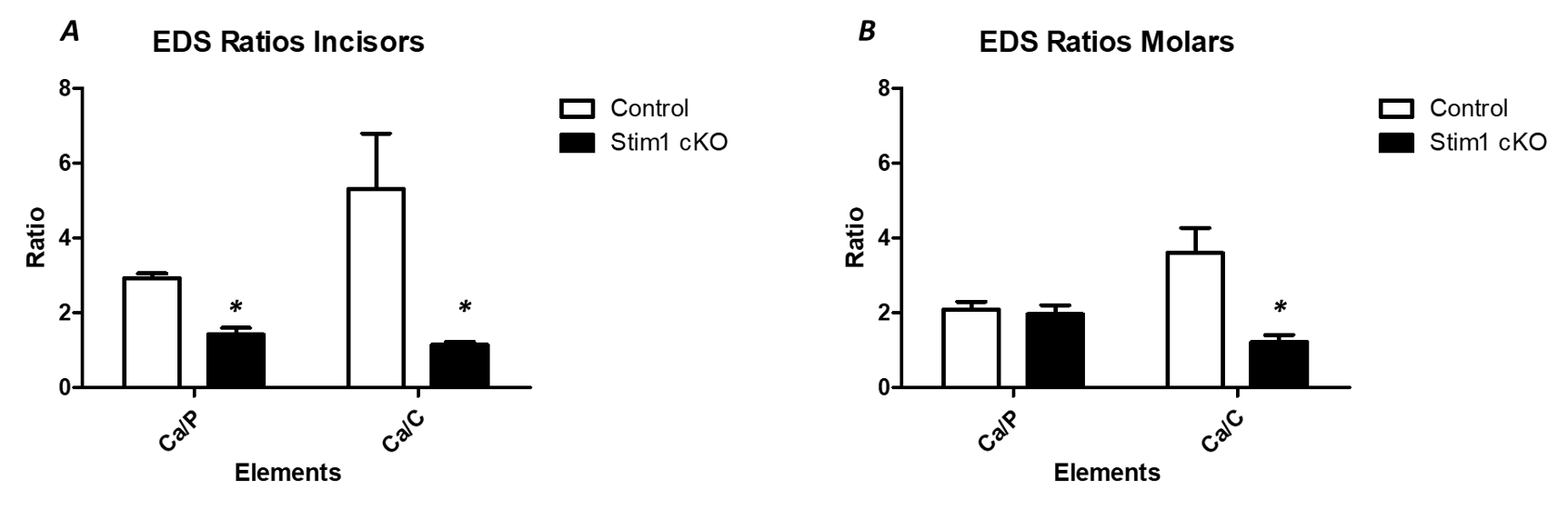

Supplement: Supplementary file 3 [file Image1.TIF]
