# Supplementary material for: Deciphering the functions of Stromal Interaction Molecule-1 in amelogenesis using AmelX-iCre mice
Source: Front Physiol. 2023 Mar 1;14:1100714. doi: 10.3389/fphys.2023.1100714 (PMC10014868; doi:10.3389/fphys.2023.1100714)
Supplement: Supplementary file 4 [file Table2.DOCX]

| [PANTHER Pathways](http://www.pantherdb.org/tools/compareToRefList.jsp?sortOrder=1&sortList=categories) | [Fold Enrichment](http://www.pantherdb.org/tools/compareToRefList.jsp?sortOrder=2&sortList=Client%20Text%20Box%20Input&sortField=foldEnrich) | [raw P value](http://www.pantherdb.org/tools/compareToRefList.jsp?sortOrder=1&sortList=Client%20Text%20Box%20Input&sortField=pval) | [FDR](http://www.pantherdb.org/tools/compareToRefList.jsp?sortOrder=2&sortList=Client%20Text%20Box%20Input&sortField=fdr) |
| --- | --- | --- | --- |
| [ATP synthesis](javascript:openDiagramWindow('/pathway/pathwayDiagram.jsp?color=1&catsInfo=true&catAccession=P02721%27);) | 8.49 | 1.40E-03 | 2.04E-02 |
| [Synaptic vesicle trafficking](javascript:openDiagramWindow('/pathway/pathwayDiagram.jsp?color=1&catsInfo=true&catAccession=P05734%27);) | 5.75 | 1.07E-06 | 4.32E-05 |
| [Metabotropic glutamate receptor group I pathway](javascript:openDiagramWindow('/pathway/pathwayDiagram.jsp?color=1&catsInfo=true&catAccession=P00041%27);) | 4.46 | 6.98E-04 | 1.12E-02 |
| [Ionotropic glutamate receptor pathway](javascript:openDiagramWindow('/pathway/pathwayDiagram.jsp?color=1&catsInfo=true&catAccession=P00037%27);) | 4.37 | 2.27E-06 | 7.30E-05 |
| [Heterotrimeric G-protein signaling pathway-rod outer segment phototransduction](javascript:openDiagramWindow('/pathway/pathwayDiagram.jsp?color=1&catsInfo=true&catAccession=P00028%27);) | 4.27 | 3.67E-05 | 7.39E-04 |
| [Metabotropic glutamate receptor group III pathway](javascript:openDiagramWindow('/pathway/pathwayDiagram.jsp?color=1&catsInfo=true&catAccession=P00039%27);) | 4.13 | 1.13E-07 | 6.07E-06 |
| [Metabotropic glutamate receptor group II pathway](javascript:openDiagramWindow('/pathway/pathwayDiagram.jsp?color=1&catsInfo=true&catAccession=P00040%27);) | 3.29 | 5.81E-04 | 1.04E-02 |
| [Cytoskeletal regulation by Rho GTPase](javascript:openDiagramWindow('/pathway/pathwayDiagram.jsp?color=1&catsInfo=true&catAccession=P00016%27);) | 3.27 | 9.37E-06 | 2.15E-04 |
| [Integrin signalling pathway](javascript:openDiagramWindow('/pathway/pathwayDiagram.jsp?color=1&catsInfo=true&catAccession=P00034%27);) | 3.19 | 3.64E-11 | 2.93E-09 |
| [Nicotinic acetylcholine receptor signaling pathway](javascript:openDiagramWindow('/pathway/pathwayDiagram.jsp?color=1&catsInfo=true&catAccession=P00044%27);) | 3.12 | 3.08E-06 | 8.25E-05 |
| [Beta2 adrenergic receptor signaling pathway](javascript:openDiagramWindow('/pathway/pathwayDiagram.jsp?color=1&catsInfo=true&catAccession=P04378%27);) | 3.10 | 1.43E-03 | 1.92E-02 |
| [Beta1 adrenergic receptor signaling pathway](javascript:openDiagramWindow('/pathway/pathwayDiagram.jsp?color=1&catsInfo=true&catAccession=P04377%27);) | 3.10 | 1.43E-03 | 1.78E-02 |
| [Muscarinic acetylcholine receptor 1 and 3 signaling pathway](javascript:openDiagramWindow('/pathway/pathwayDiagram.jsp?color=1&catsInfo=true&catAccession=P00042%27);) | 2.57 | 3.79E-03 | 3.59E-02 |
| [5HT2 type receptor mediated signaling pathway](javascript:openDiagramWindow('/pathway/pathwayDiagram.jsp?color=1&catsInfo=true&catAccession=P04374%27);) | 2.45 | 5.35E-03 | 4.54E-02 |
| [Heterotrimeric G-protein signaling pathway-Gq alpha and Go alpha mediated pathway](javascript:openDiagramWindow('/pathway/pathwayDiagram.jsp?color=1&catsInfo=true&catAccession=P00027%27);) | 2.14 | 2.43E-03 | 2.61E-02 |
| [Huntington disease](javascript:openDiagramWindow('/pathway/pathwayDiagram.jsp?color=1&catsInfo=true&catAccession=P00029%27);) | 2.02 | 1.79E-03 | 2.06E-02 |
| [Inflammation mediated by chemokine and cytokine signaling pathway](javascript:openDiagramWindow('/pathway/pathwayDiagram.jsp?color=1&catsInfo=true&catAccession=P00031%27);) | 1.69 | 4.27E-03 | 3.82E-02 |
| [Wnt signaling pathway](javascript:openDiagramWindow('/pathway/pathwayDiagram.jsp?color=1&catsInfo=true&catAccession=P00057%27);) | 1.68 | 2.55E-03 | 2.56E-02 |

**Supplemental Table 2. Panther pathway analysis of RNA_Seq data**
